# Heavy metals in the sediments of urban sinkholes in Cancun, Quintana Roo

**DOI:** 10.1038/s41598-023-34218-4

**Published:** 2023-04-29

**Authors:** Daniela Ortega-Camacho, Gilberto Acosta-González, Fernanda Sánchez-Trujillo, Eduardo Cejudo

**Affiliations:** 1 CONACYT – CICY, A.C., Unidad de Ciencias del Agua, Calle 8, No. 39, Mz. 29, S.M. 64, 77500 Cancún, Quintana Roo Mexico; 2 Universidad Tecnológica de Tehuacán, Prolongación de la 1 Sur No. 1101, San Pablo Tepetzingo Tehuacán, 75859 Puebla, Mexico

**Keywords:** Biogeochemistry, Environmental sciences

## Abstract

Soils in urban areas can accumulate heavy metals as a result of anthropogenic inputs. This research focuses on a young coastal tourist city that has been urbanized over the last 52 years and shows accelerated demographic growth and urban development. Deposition of heavy metals in soils is caused by human economic activities, which has significant implications for the environment. We evaluated heavy metal concentrations in urban sinkholes, which are sites for the natural accumulation of water and sediments. These locations also receive rainfall runoff or have been used as unregulated dumps. By performing a multistage extraction to address availability and risk, we found that Zn, Fe and Al were the predominant metals; Cu, Pb and Ni were only detected in some sinkholes. The contamination factor was high for Zn and moderate for Pb. The geoaccumulation index showed that Zn is the most abundant and available metal in urban sinkholes and the metal with the highest potential ecological risk. Between 12 and 50% of the total concentration of all metals was extracted from the organic matter phase. Correlations were found between the degree of urbanization of the city and the degree of pollution, and the trends were stronger in older sections of the city. Zn is the most prevalent element and has high concentrations. The metal concentrations in the sediments can be used as warning signs for their potential risk to environmental and human health, and these results can be compared with those of other tourist cities in karstic environments around the world.

## Introduction

Soil is a natural resource that performs vital functions for ecosystems. Owing to soil functions in the water‒soil-atmosphere continuum^1^, the deposition of heavy metals and their enrichment in soils is a global concern that is on the rise; anthropic activities mainly promote the introduction of these toxic compounds, which have important implications for humans and the environment^2^. Among the main contamination sources are mining, metalworking, agriculture, the automotive industry, and natural sources in some aquifers^3^. Heavy metal accumulation in the environment depends on a number of factors, such as soil properties (pH, redox potential, organic matter), external chemical and biological processes^1,4^ and the migration of these contaminants to water or their incorporation into the environment and food webs^5–7^.

Solid and liquid urban wastes are anthropogenic heavy-metal sources found in urban soils^8,9^. Similarly, rapid demographic growth, production of building materials (bricks and concrete), construction of shopping centers and housing developments and the excessive use of internal combustion vehicles have an increasingly negative impact on the urban environment, including the soil, air and groundwater^10^.

The dynamics of coastal cities have been modified by the construction of roads and hotels^11^. Cancun (Municipality of Benito Juárez) is the largest coastal city in the state of Quintana Roo (Mexico) and is one of the most important tourist destinations in Mexico, receiving approximately 45% of all tourists visiting Mexico^12,13^. This is a young city, and its urbanization process started in about 1970. It was conceived as a city comprising hotel and urban zones; the latter hosts managers, bureaucrats and service providers and has thus become a residential and commercial area^14^. Mass migration into Cancun reached its peak in the 1980s; it coincided with a drastic fall in wood and chicle production in Quintana Roo and a crisis in the henequen industry^15^. Between 2000 and 2010, the municipality of Benito Juárez had a population of 661,176 inhabitants and an annual demographic growth rate of 4.5%, while in 2020, the count was 911,500^16^, that is, an increase of 250,000 inhabitants in 10 years.

Despite being a recently developed city, its accelerated demographic growth has resulted in accelerated urbanization. This resulted in problems such as the inappropriate disposal of solid and liquid wastes. Urban sinkholes represent natural sites for the accumulation of water and sediments, some of which are wetlands that receive rainfall runoff or are used as unregulated dumps^17^. According to the Local Ecological Ordering Program of the Municipality of Benito Juárez^18^, in the environmental management unit designated “city”, urban population growth is allowed up to a gross average density of 80 inhabitants/ha, and spaces should comply with the recommended international indicator of 12 m^2^ of green spaces per inhabitant. Some of these green spaces consist of the urban sinkholes studied in the present research. Therefore, in this study, we contribute to existing knowledge by measuring and quantifying metals in sediments as warning signs for their potential adverse effects on environmental health from pollution in a young tourist city that has neither industrial nor mining activities.

The goal of the research was to identify contamination by heavy metals in urban sinkholes in a young city (52 years) that is growing at an accelerated rate. The results allowed us to identify zones where contamination and environmental risk are high based on the degree of urbanization. For our purposes, sediments in urban sinkholes consist of nonconsolidated materials that comprise particles originating from the soil and other sources and have been redeposited in these depressions in the landscape. The outcomes obtained here can be compared to those from other tourist cities and other urban sinkholes around the world.

## Materials and methods

### Study area

The study sites are within the city of Cancun (municipality of Benito Juárez) in the northern area of the state of Quintana Roo, Mexico. It has a subhumid climate with summer rains, a mean annual temperature of 26 °C and annual rainfall of 1229 mm, with abundant rains from June to October^19^. The geology comprises sedimentary rocks from the Carrillo-Puerto formation in the Tertiary (TmplCz-Cq); a mean 25-m thickness unit with karstification processes^20^; and the soils in the city are Lepstosol and Solonchak, with Arenosol in littoral stripes^21^. At a microscale, it is likely that the urban sinkholes in Cancun have organic soils and Gleysols given their permanent conditions of flooding and saturation^22^.

### Sample collection

We studied ten urban sinkholes, such as those described in Ref.^17^. They are classified as wetlands in palustrine systems, are depressions in the landscape with seasonal or intermittent flooding, are ovoid or round in shape, have vegetation dominated by trees that tolerate flooding, and have rocky soils with nonconsolidated substrate. The main characteristics of these urban sinkholes are presented in Table 1.

**Table 1:** General characteristics of the urban sinkholes studied in Cancun.

Site	Longitude UTM	Latitude UTM	General characteristics
A (Reg68)	518,559	2,342,260	Inside a public park. The urban sinkhole is fenced in by a chain-link mesh. In the public area, there are children’s playgrounds, benches, sidewalks and a basketball court
B (Rancho Viejo)	516,547	2,344,597	A green area located at the municipal border between Benito Juárez and Isla Mujeres, which is at the crossroads of two heavy-traffic roads and in front of a service station. Clandestine dump
C (Reg230)	515,681	2,341,566	A public park with a central urban sinkhole and three man-made ponds. In the park, there are benches, sidewalks, a basketball court, open-air fitness equipment and children’s playgrounds. The park is utilized for ludic activities (Scout group)
D (Reg217)	512,891	2,340,704	A green area contiguous to a soccer field. A leisure area and pedestrian crossing
E (Reg100)	513,438	2,339,681	A sinkhole wetland with a semiflooded cave; visible water is present through almost the entire contour. Depression of approximately 6 m. It is within a public park that has open-air fitness equipment and a sidewalk in the middle of the outer perimeter
F (Reg97)	513,422	2,338,657	A sinkhole wetland in a heavy-traffic rotary intersection. Depression of approximately 4 m. It has been turned into a clandestine dump of solid wastes, electronic appliances and outdated drugs. Road accidents are frequent in this location
G (Reg510)	514,093	2,337,107	A sinkhole wetland inside a secondary school. It is fenced in by a chain-link mesh to prevent access
H (Reg524)	514,929	2,336,684	A sinkhole wetland in the median strip of a road with heavy traffic. It has a sidewalk built by residents around most of its entire perimeter. In the zone, there is accumulation of commercial and household solid wastes
I (Reg523)	515,310	2,336,893	A sinkhole wetland turned into a park by a housing developer. It is used by residents for leisure; it has a small cave with permanent water and fish
J (Parque Kabah)	516,948	2,338,062	A sinkhole wetland inside a State Natural Protected Area

Composite samples were taken by means of directed sampling according to the accessibility of the locations. Three subsampling points were chosen, each place was superficially cleaned from litterfall, and a “V” excavation was made with a plastic tool at a depth of at least 20 cm. Leaves, twigs, and visible roots were manually removed, and the sediment was placed in a large clean plastic vat. This procedure was carried out at each subsampling point. Once all the subsamples were collected, they were mixed in a plastic vat, and the composite samples from each site were stored in a previously labeled airtight bag. Because excessive amounts of water were present in the samples, they were left to dry at ambient temperature and later in a drying oven on plastic trays for 48 h at 60 °C. Once dry, leaves, roots and rocks were removed again, the first sieving was performed with a basic approximately 5-mm plastic mesh. The retained material was stored in plastic bags as coarse material. Later, the material from the first sieving was passed through another mesh (≈ 2 mm) and was designated the fine material. To accomplish better homogenization, the fine material was ground with a porcelain mortar and pestle and sieved again with a 2-mm mesh; this was the material used for the sequential extraction.

### Sediment sample processing

**Figure 1:**
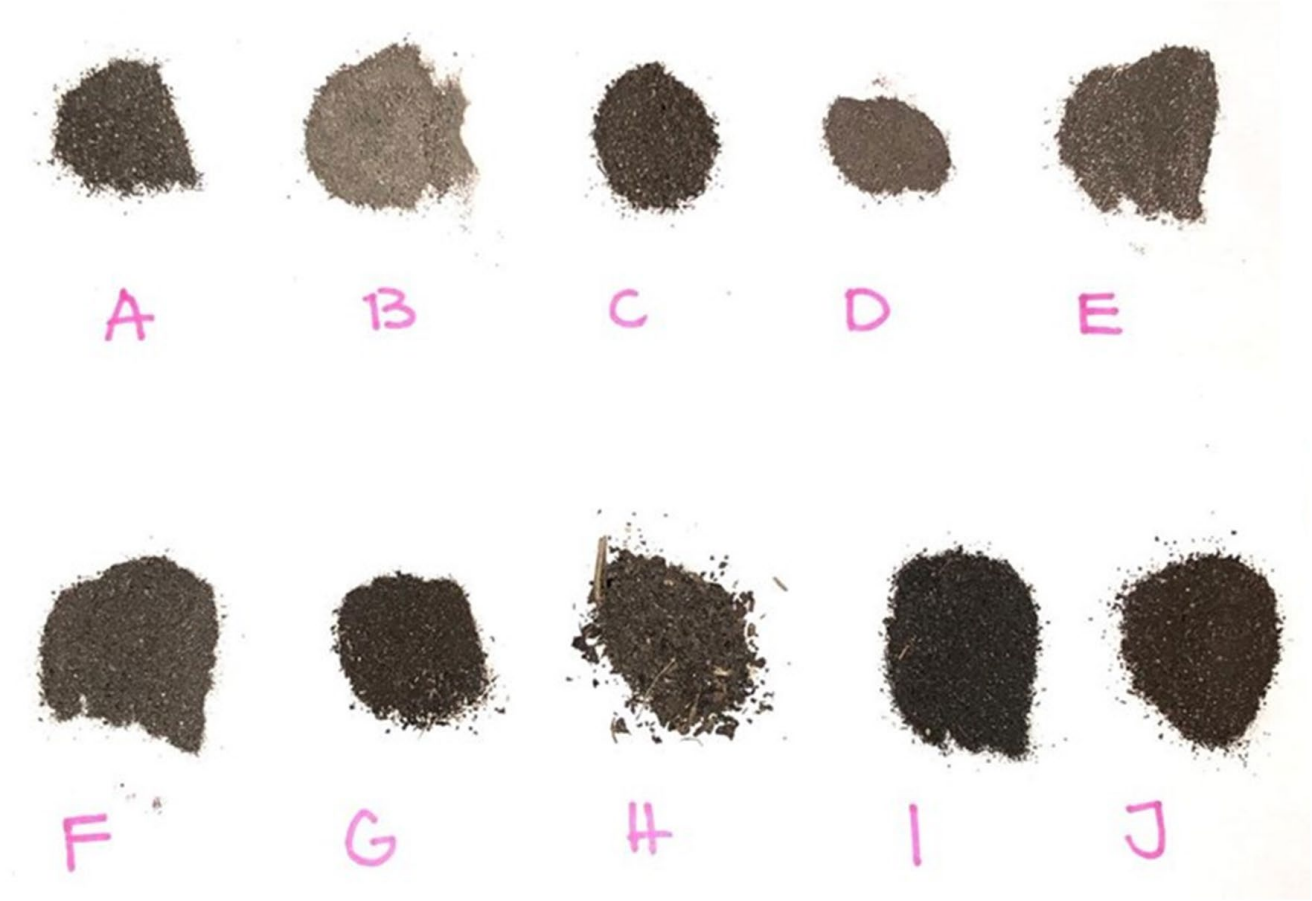
Dry color photograph of the soils in the study (particle size ≈ 2 mm).

To identify color, two 5-g portions of each sample were placed in laboratory watch glasses. Using Munsell’s color digital chart, one of the portions was used to identify “dry color” (Fig. 1), while the other was moistened with Milli-Q water and was used to identify “wet color”. Hue, value and chroma were integrated, and a name was assigned to the value. This procedure was repeated for wet colors (water was added up to the saturation value), and the results were recorded. Additionally, pH, electrical conductivity (dS/cm), ferrous iron content (mg Fe^2+^/kg soil dry weight) and organic matter content (%) were measured in the samples. pH was measured with a potentiometric method^23^; electric conductivity (soil‒water 1:2) was measured with a glass electrode and a Laqua PH1100 potentiometer (Horiba Scientific^®^). Ferrous iron concentration was determined using a method derived from the analysis of Fe^3+^ in water by Ref.^24^, in which α,α-dipyridyl was used as a colorimetric indicator of ferrous iron in the analysis of soil aqueous solutions. Values in units of mg/L were converted into mg/kg (dry weight) based on the initial sediment mass used for the extraction. Organic matter was measured following Ref.^25^.

### Sequential extraction

Multistage extraction allows differentiating the mobile fractions of residuals and characterizing the most labile fractions^4^, offering complementary information to ascertain existing environmental risk^26^. The sequential extraction method described by Ref.^27^ was carried out, except for the phase associated with iron and manganese oxides, and a phase called water-soluble was added. One gram of fine material was transferred to a 50 mL centrifuge tube to carry out the extraction. Between each extraction step, the supernatant of the sample was obtained by means of decantation and was aided by centrifugation of the tube in a Thermo^®^ IEC Centra CL2 machine (3000 RPM, 10 min). The solid material in the tube was then used in the following extraction step. The final volume of the supernatant of each stage was set to 30 mL, and this volume was reached with Type I water (resistivity < 0.2 mΩ; electric conductivity < 5.0 µS). The extraction phases were as follows:

1. Water-soluble: agitation with 15 mL of Type I water for 30 min.
2. Exchangeable: agitation with 10 mL of 1 M MgCl_2_ for two hours.
3. Carbonate-bound: agitation with 10 mL of 1 M CH_3_COONa for five hours.
4. Organic matter-bound: an amount of 3 mL HNO_3_ 0.002 N was added and allowed to react to completion. Then, 8 mL of H_2_O_2_ was slowly added (pH 2 adjusted with more HNO_3_ 0.002 N), and the suspension was agitated at a constant temperature (85 °C) for five hours. Finally, 3 mL of H_2_O_2_ was added (pH 2 adjusted with HNO_3_), and the suspension was kept at 85 °C for three hours. After this, 5 mL of 3.2 M CH3COONH4 was added to 20% v/v HNO3.
5. Residual: carried out according to the method EPA 3052^27^. The residue of stage four was added to 10 mL of concentrated HNO_3_ (1:1) and heated at 90 °C with reflux for 15 min. The sample was left to cool, 5 mL of concentrated HNO_3_ was added, and the suspension was kept at 90 °C for 30 min. Then, 2 mL of Type I water and 3 mL of 30% H_2_O_2_ were added up to minimal effervescence. Finally, the sample was heated (90 °C) for two hours to reduce its volume to a final value of 30 mL.

Additionally, total sediment digestion was also carried out according to Ref.^28^ following the same methodology as that for the residual fraction.

### ICP‒OES analysis

All the aqueous phases from the sequential extraction and total digestion were analyzed by ICP‒OES (Perkin Elmer Optima 8000). Quality control was achieved during quantitative analysis by means of blanks and calibration curves based on a commercially available standard (ICP TraceCERT^®^ Multielement Standard Solution 6). The instrument detection limit was 20 μg/L. In both the sequential extraction and total digestion, we used the certified reference material San Joaquin Soil (NIST^®^ SRM^®^ 2709a) with a minimum recovery of 72.5% for Al and a maximum recovery of 115% for Cu.

### Ecological risk assessment

With the results from the sequential and total quantifications, we assessed the following indices. The contamination factor^29^, CF*,* identifies anthropogenic contributions; it is widely utilized to assess soil contamination. It is calculated from the ratio of metal concentration in soil and the metal’s mean concentration in the Earth’s crust:

1 $$\mathrm{CF}= \frac{{\mathrm{C}}_{\mathrm{i}}}{{\mathrm{B}}_{\mathrm{i}}}$$

where ${\mathrm{C}}_{\mathrm{i}}$ corresponds to metal *i* concentration in soil, and ${\mathrm{B}}_{\mathrm{i}}$ is the metal geochemical background value. The contamination factors are classified as follows: 0 = no contamination; 1 = none to medium; 2 = moderate; 3 = moderate to heavy; 4 = contaminated; 5 = heavy to very heavy; 6 = severe contamination. The B_i_ values used in this study are Zn = 70 mg kg^−1^; Fe = 3800 mg kg^−1^; Al = 4200 mg kg^−1^; Cu = 55 mg kg^−1^; Ni = 20 mg kg^−1^ and Pb = 9 mg kg^−1^^30^.

The degree of contamination^29^, C_D_, represents the sum of CF for all metals per site and is classified into four degrees: C_D_ < 6 = low contamination degree; 6 < C_D_ < 12 = moderate; 12 < C_D_ < 24 = high; and C_D_ > 24 = very high.

The pollution load index^31^, PLI, is the geometric mean of the CF values:

2 $$\mathrm{PLI}=({\mathrm{CF}1 \times \mathrm{CF}2 \times \mathrm{ CF}3 \times ..\times \mathrm{ CFn})}^{1/\mathrm{n}}$$

Values over 1 suggest the existence of contaminated soil, whereas values ≤ 1 are associated with no pollution.

The enrichment factor, EF, is calculated on the basis of the abundance of a metal in relation to its mean abundance in the Earth’s crust. The following equation is used:

3 $$EF={\left(\frac{X}{Y}\right)}_{sample}/{\left(\frac{X}{Y}\right)}_{crust}$$

where X is the concentration of the metal and Y is the concentration of a reference metal. The reference metal is usually Fe or Al owing to their natural abundance. In this case, the metal used was Fe. Reference values for each metal in the Earth’s crust were the same as those used for the calculation of CF.

The geoaccumulation index I_geo_^32^ is a classification system that identifies the chemical concentration of a metal related to the Earth’s crust reference values:

4 $${\mathrm{I}}_{\mathrm{geo}}= {\mathrm{log}}_{2}\frac{{\mathrm{C}}_{\mathrm{i}}}{{\mathrm{B}}_{\mathrm{i}}\mathrm{x}1.5}$$

where ${\mathrm{C}}_{\mathrm{i}}$ is the concentration of the metal and ${\mathrm{B}}_{\mathrm{i}}$ is the reference concentration. A factor of 1.5 is introduced to include certain variations caused by the matrix^32^. There are seven geoaccumulation categories: 0 = no contamination; 0–1 = no contamination to moderate contamination; 1–2 = moderate contamination; 2–3 = moderate contamination to high contamination; 3–4 = heavy contamination; 4–5 = heavy contamination to extreme heavy contamination; and 5 = extreme heavy contamination.

Finally, to evaluate the associated ecological risks, we estimated the single factor potential ecological risk E^i^_r_^29^ and the comprehensive potential ecological risk index RI^33^. The single factor potential ecological risk estimates the contamination by an element by considering the potential toxicity (T^i^_r_) and the concentration of each element in the sample (C^i^_m_) and the background concentration (C^i^_n_). The values of the toxicity coefficients T^i^_r_ used were previously reported^32,34,35^.

5 $${E}_{r}^{i}=\frac{{T}_{r}^{i} \times {C}_{m}^{i}}{{C}_{n}^{i}}$$

The comprehensive potential ecological risk index is the sum of E^i^_r_

6 $$RI={\sum }_{i=1}^{n}\frac{{T}_{r}^{i}\times {C}_{m}^{i}}{{C}_{n}^{i}}$$

The single factor potential ecological risk is E^i^_r_ ≤ 20—mild, E^i^_r_ = 20 to 40—moderate, E^i^_r_ = 40–890 relatively strong, E^i^_r_ = 80–160—strong and E^i^_r_ ≥ 160—very strong. The RI is classified as RI ≤ 110—mild, 110 ≤ RI ≤ 220—moderate, 220 ≤ RI ≤ 440—relatively strong and RI > 440—strong^33^.

A nonmetric multidimensional scaling (MDS) ordination was used to represent sampling locations in two-dimensional space in terms of the concentration of heavy metals in the sediment and grouped by the degree of contamination. The similarity matrix for nonmetric MDS was calculated using Bray‒Curtis similarity; MDS was conducted using PRIMER 6.

## Results

The sediment in the urban sinkholes under study was largely grayish–blackish in color, presumably due to the high content of organic matter (Table 2). The pH values in the samples were close to neutrality (6.61–7.33). Electric conductivity (dS/cm) was quite variable; the lowest value of 0.74 dS/cm was observed at site B, while the highest value of 4.54 dS/cm was observed at site I (a sinkhole turned into a park). Finally, ferrous iron displayed values that varied between 120 and 270 mg/kg.

**Table 2:** Description of the soil from the urban sinkholes in the study.

Site	Dry color	Description	Wet color	Descriptive	pH (water)	pH (KCl)	EC [dS/cm]	Fe^2+^ [mg/kg d.w]	Eas. Oxi. Org. C (%)	O.M. (%)
A	10 YR 5/1	Gray	10 YR 2/1	Black	6.61^a^	6.61^a^	4.17	184.4 ± 6.2	21 ± 1	62 ± 1
B	10 YR 6/1	Gray	7.5 YR 4/1	Dark gray	7.3^a^	7.1^a^	0.74	275.2 ± 23	11 ± 1	31 ± 1
C	10 YR 4/1	Dark gray	10 YR 2/1	Black	6.79^a^	6.57^b^	0.79	202 ± 19	14 ± 1	42 ± 3
D	10 YR 5/1	Gray	10 YR 2/1	Black	7.23^a^	7.11^c^	1.4	256 ± 33	7 ± 1	20 ± 3
E	10 YR 5/2	Grayish brown	10 YR 2/1	Black	7.21^a^	7.08^a^	1.55	247.2 ± 8.3	12 ± 2	36 ± 6
F	10 YR 4/1	Dark gray	10 YR 2/1	Black	7.32^a^	6.91^a^	1.63	222.4 ± 6.2	5 ± 1	16 ± 3
G	10 YR 3/1	Very dark gray	7.5 YR 2/1	Black	7.17^a^	7.1^a^	3.61	225.2 ± 6.2	29 ± 2	85 ± 6
H	7.5 YR 3/1	Very dark gray	7.5 YR 2/1	Black	7.41^a^	7.24^a^	2.68	152 ± 2.1	11 ± 2	31 ± 6
I	10 YR 2/1	Black	10 YR 2/1	Black	6.6^a^	6.97^a^	4.54	119.6 ± 10	8 ± 1	24 ± 3
J	7.5 YR 4/1	Dark gray	7.5 YR 2/1	Black	7.06^a^	6.88^a^	1.73	138.8 ± 17	5 ± 1	16 ± 3

pH: *a* Neutral, *b* Acidic, *c* Basic. EC—electrical conductivity; Eas. Oxi. Org. C—Easily oxidized organic carbon; O.M.—Organic matter. Fe^2+^, Eas. Oxi. Org. and % O.M. mean value ± 1 s.d

**Figure 2:**
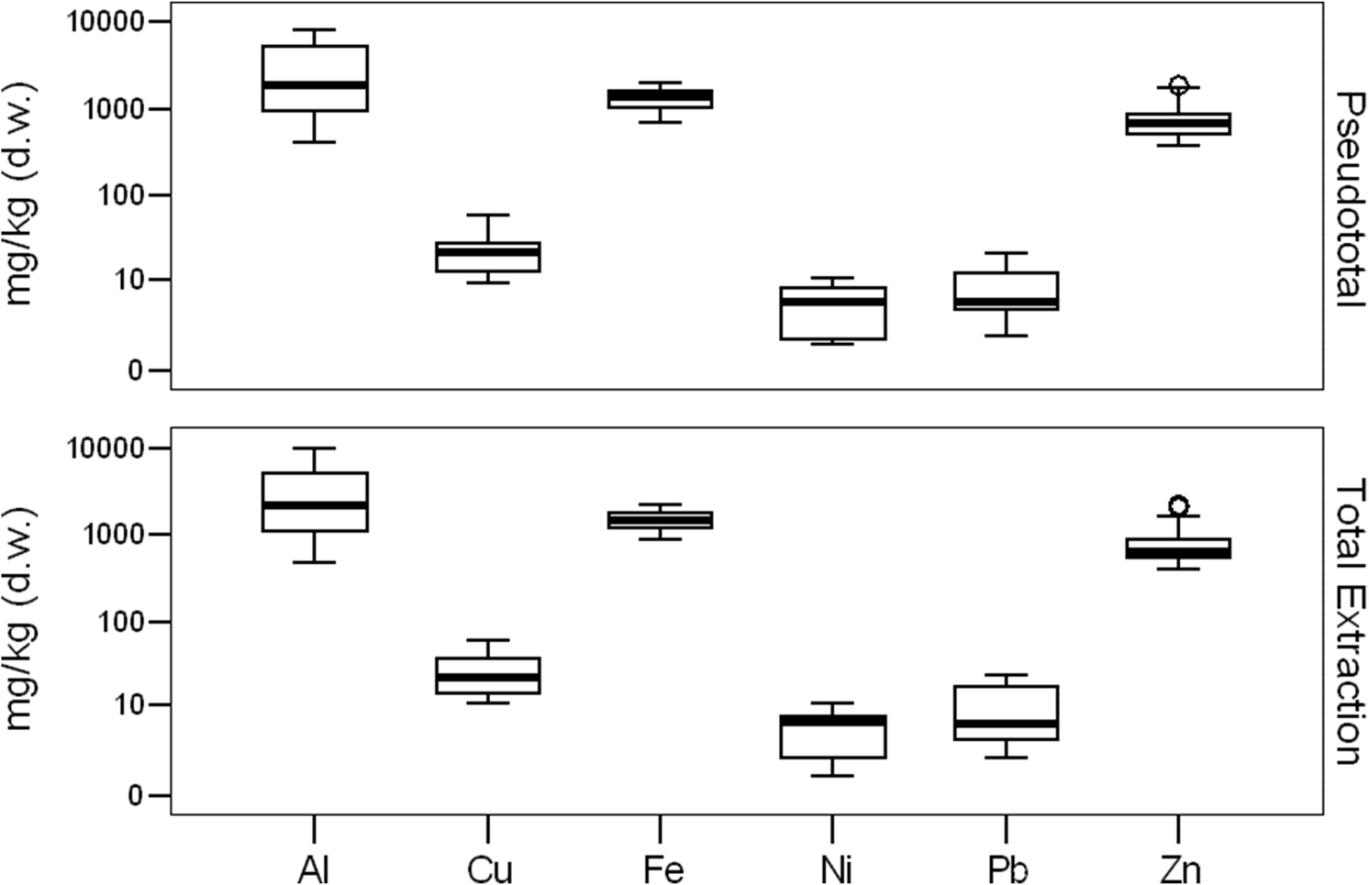
Metal concentration in sediments (mg/kg soil, dry weight) in the urban sinkholes in the study.

Zn, Fe and Al were the predominant metals in the soil, with mean concentration values of 873 mg/kg, 1502 mg/kg and 3184 mg/kg, respectively, followed by Cu (26.989 mg/kg). Pb was only quantified in six of the sites within an interval from 1.76 to 23.19 mg/kg (Fig. 2). In a similar manner, Ni was not quantified in all the sites. It was found in only seven out of ten and at low concentrations between 1.19 and 8.8 mg/kg.

**Figure 3:**
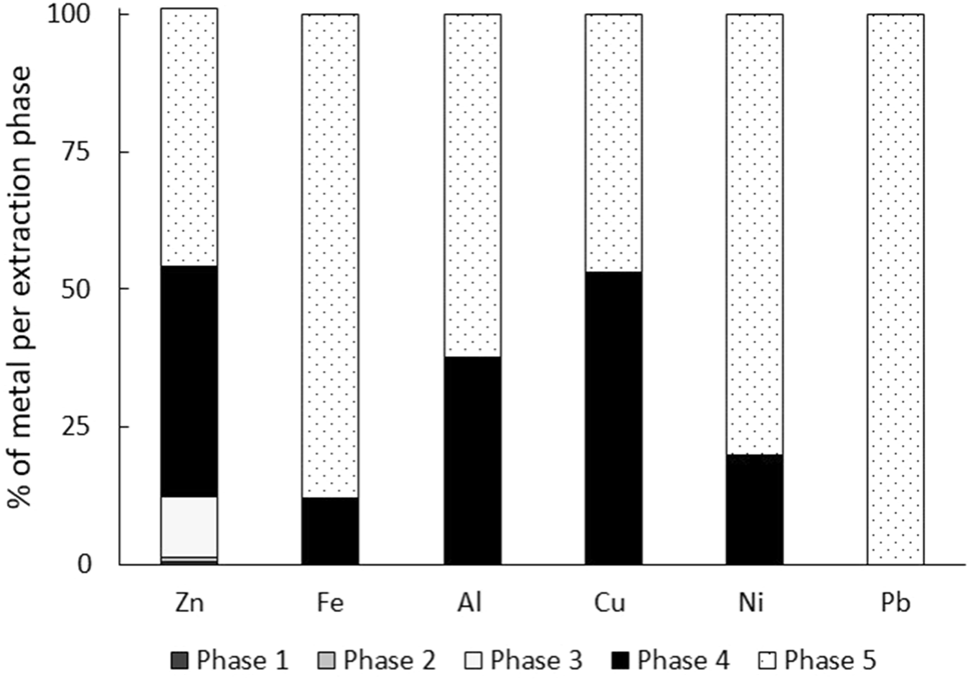
Percentage of metal in each extraction phase in the sediments from the urban sinkholes in this study. Phase 1—Water soluble; Phase 2—Exchangeable; Phase 3—Carbonate bound; Phase 4—Organic matter bound; Phase 5—Residual.

The only metals extracted in the first three phases were Zn and Al; in terms of percentage, the water-soluble and exchangeable phases for these metals were under 1%. For Zn, the carbonate-bound phase (3) only extracted 10% of this metal. Figure 3 shows the extraction percentage for each metal based on the phase of the sequential extraction process as a proportion of the means of each metal for all the sites.

Pb was the only metal extracted solely in the residual phase at all the sites where it was quantified. In the residual phase, 80% of Fe and Ni was extracted, followed by Al at 60%. In the case of Zn and Cu, in both the residual phase and the organic-matter bound phase, between 40 and 50% of these metals was extracted. In the OM phase, all metals were extracted within a range from 12 to 50%, which points toward the affinity of the studied metals to this phase. The full results of the extractions by element and site are presented in Table S1.

### Indices

**Figure 4:**
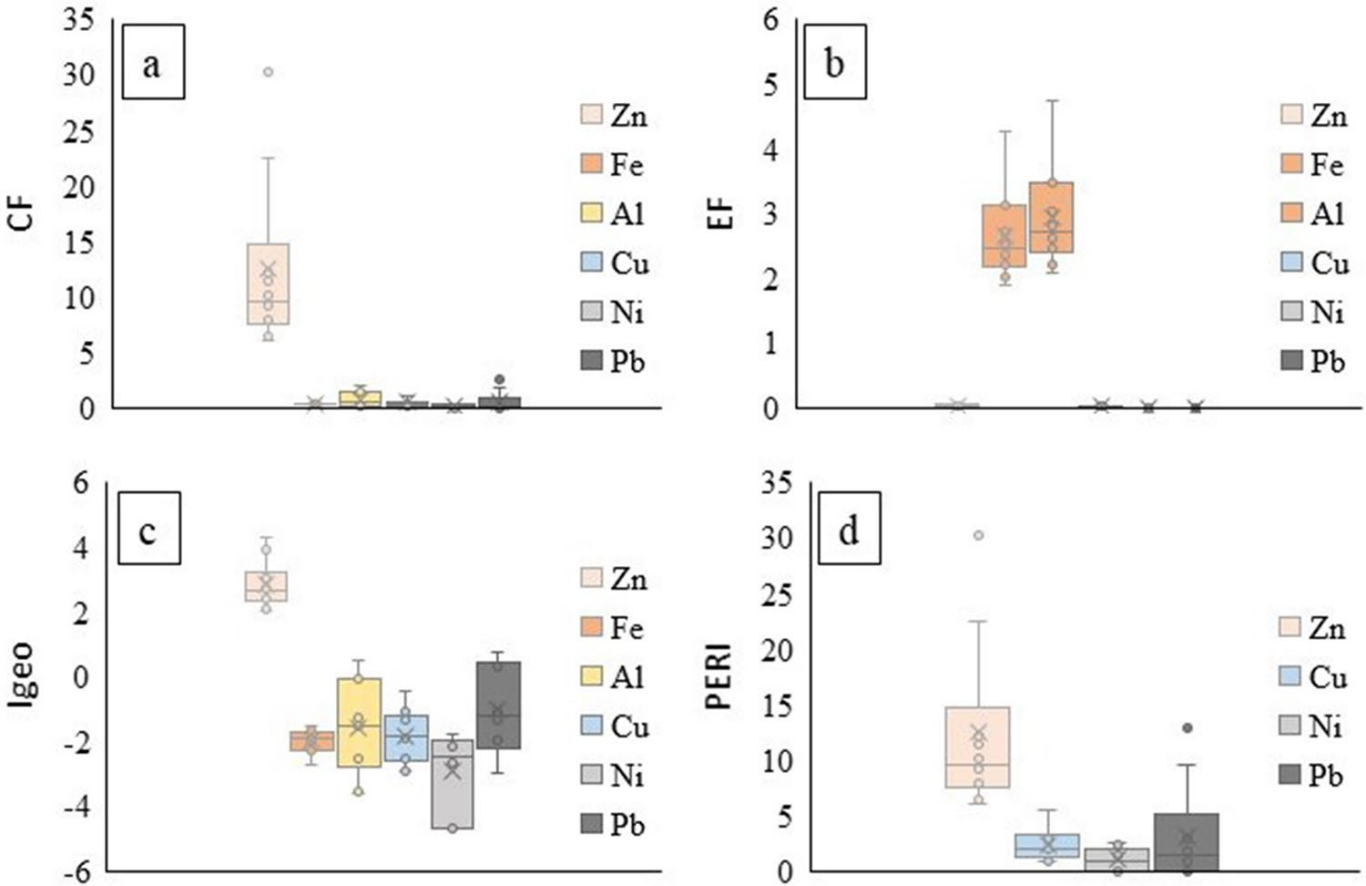
Soil indices assessed in the 10 urban sinkholes in the study in Cancun. (**a**) Contamination factor (CF), (**b**) enrichment factor (EF), (**c**) geoaccumulation index (I_geo_) and (**d**) potential ecological risk index (PERI).

**Figure 5:**
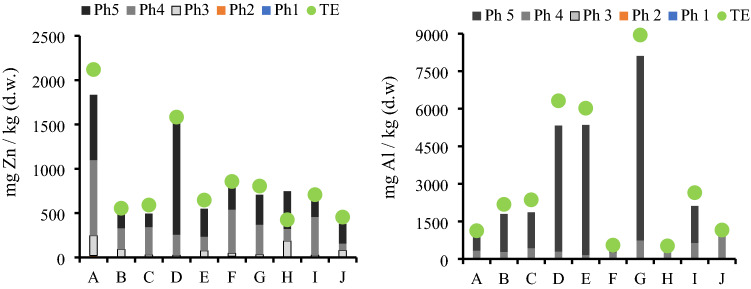
Zn (*left*) and Al (*right*) concentrations (mg/kg soil, dry weight) in the urban sinkholes in the study in Cancun. *Ph*-Phase (1 to 5) *TE—*Total extraction.

The values of the indices assessed in the 10 urban sinkholes are shown in Fig. 4 (complete results in Table S2). The contamination factor (CF) was strong for Zn, moderate for Pb and Al at two sites, and medium for Al, Cu and Pb at three sites. Fe and Ni showed no risk in any urban sinkhole. The enrichment factors (EFs) indicated that for the 10 sites, the sources of Zn, Cu, Ni, and Pb were natural, whereas Fe and Al had moderate enrichment factors. The geoaccumulation index (I_geo_) indicated that the soils were moderately to heavily contaminated with Zn in all the study sites, and Zn had the highest potential ecological risk (PERI, Fig. 4d). Because of the importance of Zn and Al, the concentrations of these metals in each urban sinkhole are shown in Fig. 5.

The pollution index (PLI) suggests that the sediments from all the urban sinkholes were polluted (Table 3). The contamination degree (C_D_) was estimated with and without the influence of Zn since it was the most abundant metal in all urban sinkholes. When Zn was included, the C_D_ was moderate at three sites, considerable at five sites, and very high at two sites (A and D). When Zn was not included, all urban sinkholes had low values (< 5). This last finding suggests that Zn is an element with a high presence in the soils of these urban sinkholes and the one with the most influence on the indices evaluated.

**Table 3:** Pollution load index (PLI) and degree of contamination (C_D_) for each of the urban sinkholes under study in Cancun.

Site	PLI	C_D_	C_D_ (w/o Zn)
A	1.80	34.57	4.28
B	1.46	9.52	1.60
C	1.48	10.36	1.92
D	1.72	25.78	3.37
E	1.52	12.29	3.05
F	1.59	16.19	3.95
G	1.56	14.42	2.92
H	1.39	7.14	1.07
I	1.51	11.68	1.56
J	1.40	7.44	0.92

C_D_ (w/o Zn)—Degree of contamination without zinc.

The single factor ecological risk indicates that Al, Ni and Pb pose a mild degree of risk, Cu has a moderate degree of risk in two sinkholes (A and F) and Zn is the metal of highest concern, from moderate to strong (Table 4). The comprehensive potential ecological risk index concurs with the single factor ecological risk, mild for Al, Ni and Pb, moderate for Cu and strong for Zn. It is important to emphasize that in terms of carcinogenic risk, both Cu and Zn showed a high risk index.

**Table 4:** Single factor potential ecological risk (E^i^_r_) and comprehensive potential ecological risk index RI evaluated for each of the urban sinkholes under study in Cancun.

E^i^_r_	Zn	Fe	Al	Cu	Ni	Pb
A	106.0	*n.a*	0.5	20.0	0.4	12.9
B	27.8		1.0	17.8	0.0	1.0
C	29.6		1.1	8.4	0.3	1.9
D	79.2		3.0	16.8	0.3	3.0
E	32.4		2.9	11.2	0.1	3.6
F	42.9		0.3	30.4	0.4	9.5
G	40.3		4.3	7.4	0.0	0.0
H	21.3		0.2	5.7	0.2	0.0
I	35.4		1.3	12.2	0.1	0.0
J	22.8		0.6	5.5	0.0	0.0
RI	437.3	–	15.2	135.2	1.8	31.9
RI*	2186.5			675.8		

RI * Carcinogenic risk. Fe toxicity coefficient not available (*n.a*.); risk factors were not computed.

**Figure 6:**
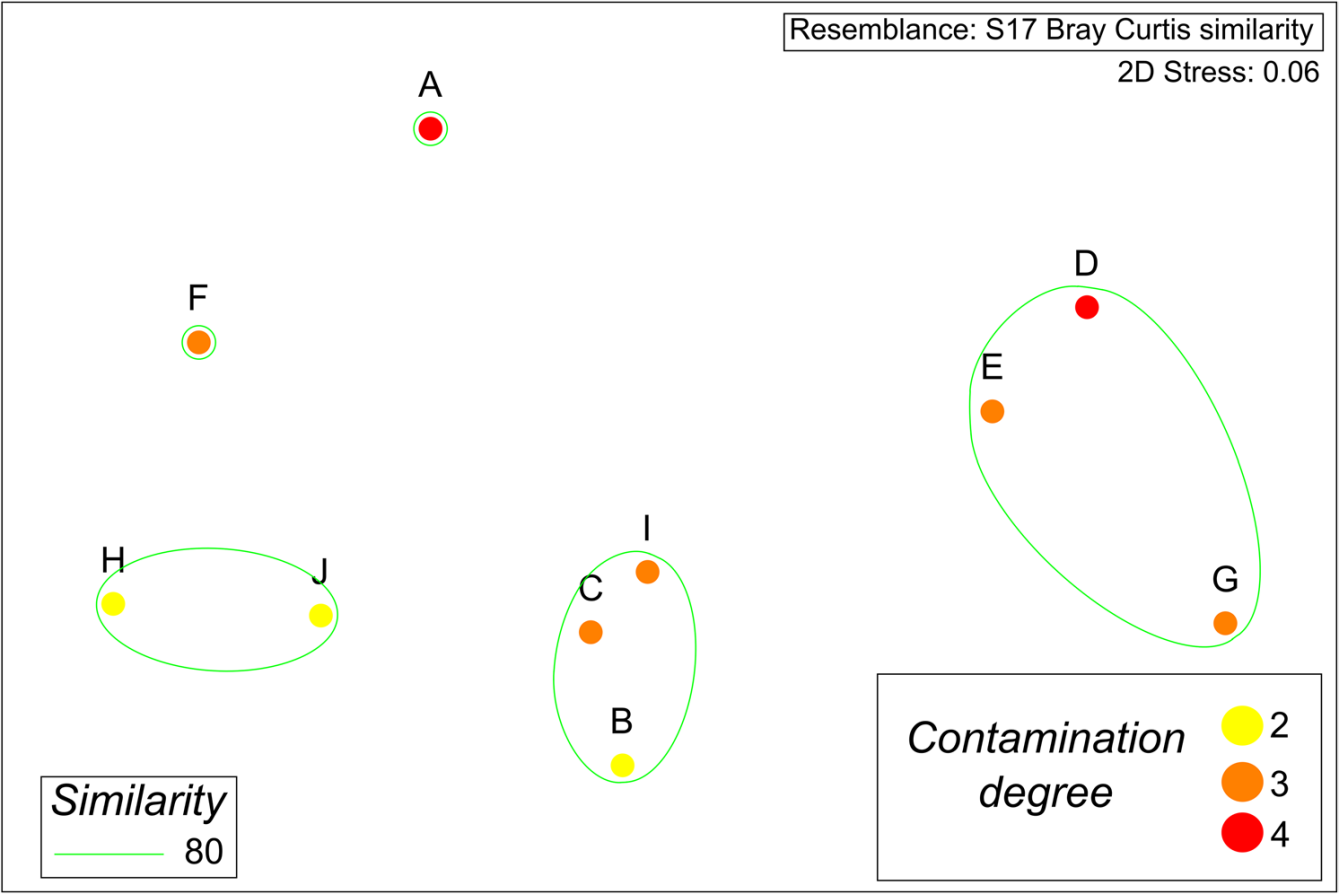
Nonmetric multidimensional scaling (MDS) ordination used to represent the urban sinkholes in study in terms of the concentration of heavy metals in the sediments.

**Figure 7:**
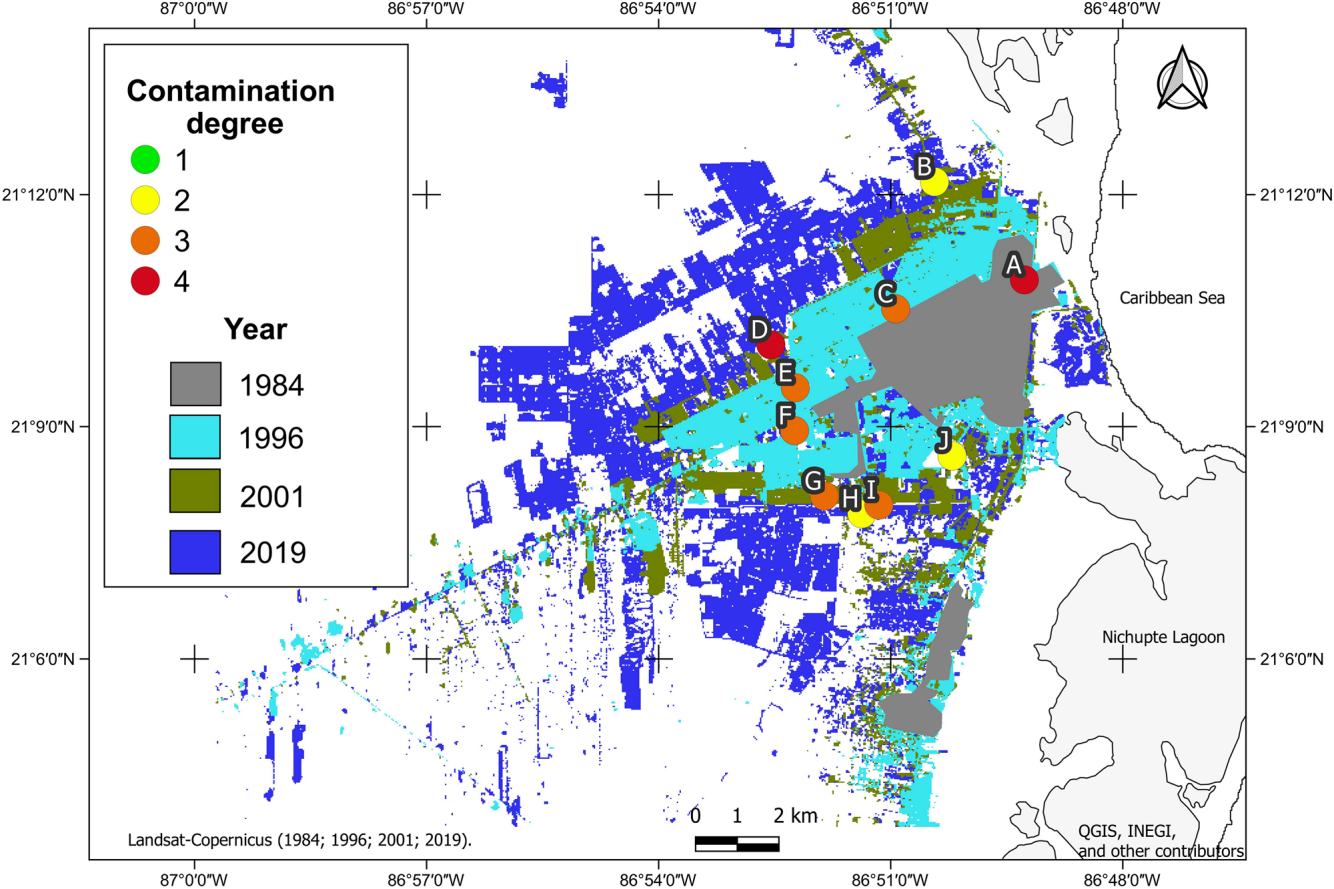
Degree of contamination of the sediments in the urban sinkholes in the study relative to urbanization time. The oldest areas in the city (dark gray) comprise sites with high degrees of contamination. Map generated with QGIS 3.14.1 [https://www.qgis.org/en/site/]; vectorial layers from INEGI [https://www.inegi.org.mx]. Landsat TM satellite images from 1984, 1991, 2001, and 2019 (with less than 30% cloud cover; http://earthexplorer.usgs.gov/) were obtained and cropped (area of interest) and a supervised classification was applied with QGIS version 3.14.1, with two classes: City and Other land uses.

The zones that have been urbanized for long periods (1984–1996) display higher contamination levels (4 and 3) than more recently urbanized zones (2001–2019), which have lower degrees of contamination. Additionally, the nonmetric multidimensional scaling (MDS) ordination depicted the grouping of sinkholes based on the concentration of heavy metals (Fig. 6). We noticed that sites A and F are separated from the rest because of the elevated concentration of metals, with sinkhole A being located in the oldest part of the city and sinkhole F being located in one of the main streets in the city, completely surrounded by roads (inside a roundabout). The groups with H and J correspond to sites with low impact from urbanization; the former is a park in a recent development, and J is a sinkhole inside a State Natural Protected Area. Finally, the two other groups seem to be aligned in a vertical array, probably reflecting the inland progression of urbanization. These results suggest that despite being a recently developed city (52 years), Cancun has places with high degrees of contamination, which are clearly in the oldest parts of the city (Fig. 7). The sites with contamination indices between 3 and 4 are those that have measurable concentrations of Pb and Ni (the other sites do not have these metals above the quantification limit).

## Discussion

The dominance of Leptosol soils in the study area suggests that they are the main source of sediments in the urban sinkholes. Leptosols are thin, poorly developed soils with large amounts of calcareous material^36^, which, under pH neutrality conditions, favor the immobilization of metals in carbonates^37^.

The reduction from ferric (Fe^3+^) to ferrous iron (Fe^2+^) is particularly important in the diagnosis of anaerobic conditions in saturated soils. The quantification presented in this study hints at reducing conditions in urban sinkholes. Fe and Cu were released from the fraction associated with organic matter since these two metals are frequently found as oxides or hydroxides in clays and humus^38^. The high content of organic matter in urban sinkholes may be one explanation for the accumulation of Cu at high risk levels.

Pb and Ni are the only metals considered in the Mexican regulations^39^ that define the concentration of heavy metals in soil. These metals are not present at levels that surpass the limits defined for residential or commercial soils (Pb 400 mg kg^−1^ and Ni 1600 mg kg^−1^). Based on this classification, the soil is not considered to be polluted with metals.

Pb, Ni, and Fe were extracted at high proportions or were exclusively present in the residual phase, which suggests that these metals are closely associated with oxide structures (mostly for Fe) and silicate minerals^40^. Al was detected in all the phases in most sinkholes, although the percentages were different; its extraction order was as follows: water-soluble (0.11%) < interchangeable (0.122%) < carbonate bound (0.3%) < organic-matter bound (37.37%) < residual (62.16%). Al is slightly affected by erosion and weathering processes, which is the reason for its relative abundance in the residual and crystalline phases^41^.

For Cu, 53% of this metal was extracted in the organic-matter bound phase, whereas 46.9% was extracted in the residual phase. In this study, Cu was the only metal that was not extracted largely from the residual phase but in the organic matter-bound phase; this behavior agrees with other authors’ findings^42^. Cu is more abundant in this phase owing to the numerous compounds it produces with organic matter in soils^1^.

At some sites, Zn was also detected in all phases. The order of extraction for this metal is as follows: water-soluble (0.37%) < interchangeable (0.982%) < carbonate bound (11.13%) < organic-matter bound (41.6%) < residual (46.9%). It is important to stress that in early extraction phases, Al prevailed in most of the sites, but this was not the case for Zn, which was only quantified in three sites in the early extraction phases. Zn was released in the early extraction stages, as this metal has the tendency to be absorbed in carbonates and oxides, since dissolution/precipitation reactions retain metallic ions in crystalline matrices^1^. Similarly, the high prevalence of Zn in the organic-matter bound phase may be due to the tendency of this material to be absorbed to organic-type molecules^43^. Although there are no clear trends in the behavior of this metal, it has been observed that as its concentration increases, it becomes distributed in the residual and organic phases, as was found in this work^42,44^.

Nonresidual phases are the most mobile metal phases, as they may be more readily available for humans and plants, owing to the conditions in which they are released (oxidants, organic matter)^45^. Based on the results of the present study, the most bioavailable metals are Zn, Cu, Al, and Fe. Since the residual phase is not solubilized for long periods of time under natural conditions, Pb and Ni would be the least available metals in the environment^46^.

To verify the above, CF was calculated for sequential extraction as a contamination index. The values over 1 correspond to Zn and Al, which are considered more available for mobilization due to the proportion in which they were found in the nonresidual phases. Cu, Ni, and Pb do not pose a threat because they are largely retained in the residual phase, which means that they will not be easily released under environmental conditions. A metal retained at the surface of sediments by complexation, precipitation or sorption will be dissolved rapidly, and because of this, it would pose a higher toxicity risk than if it was embedded in the soil’s crystalline structure^1^.

Zn can be considered a contaminating metal according to all the indices evaluated. Pb, Al and Cu seem to be in the early contamination stages; they might become a problem at some time in the future should the accumulation trends continue. With the exception of Zn, the metals show low to moderate potential ecological risk, and some of them seem to be present in natural quantities based on the mean composition of the limestone rock^30^. As the indices show, metal enrichment occurs not only due to their geological presence but also due to external contributions. In this case, the apparent contributions are from solid urban wastes, which have only been generated and accumulated 50 years but already represent a latent and potentially increasing risk for the city of Cancun.

Owing to the high concentration and prevalence of Zn, the estimation of CD with and without this metal implies that its presence in the sediments of urban sinkholes clearly comes from allochthonous sources, i.e., produced by anthropogenic action. Pb, Zn, and Cu are the most abundant metals in sites with contaminated sediments, and their presence indicates risk of groundwater contamination^3^.

The typical Zn concentration in soil is between 10 and 300 mg/kg^47^. High Zn concentrations (higher than 1000 mg/kg) are found in soils whose minerals are composed of this metal^48^. Soils in urban areas are commonly contaminated with Pb, Zn, Cd and Cu from vehicles, paint and other urban sources^49,50^. Zn is likely to be found in areas with vehicular traffic, as this metal is an additive that makes up the materials of tires and may be released by tire abrasion^50^. High Zn concentrations may result from the numerous applications of this metal in the electronic, chemical, electric, medical and cosmetic industries^51^.

The typical concentration of Pb in surface soils around the world is a mean of 32 mg kg^−1^ and has a range of 10–67 mg kg^−1^^52^. The main sources of Pb emissions into the environment are foundries, metal processing, the recycling of lead-acid batteries, mining, and atmospheric contamination from the use of leaded fuels^53^. Specifically, site A (Reg68), which corresponds to the oldest area of Cancun, had the highest concentration of Pb. This could be due to the use of leaded gasoline during the early urbanization of the city, as leaded gasoline was permitted before the 1990s^53^. Fortunately, this metal concentration has decreased in recent years as a result of governmental regulations around the world.

The presence of metals such as Pb, Cu, and Zn may be due to the heterogeneity of solid wastes (including electronic waste), which are related to the release of these metals into the environment^54^. Urbanization does not contribute as much as conventional industries; however, urban areas are permanent pollution sources, continually releasing contaminants^55^, such as urban dust^56^. Urban dust is a heterogeneous mixture of compounds, in which heavy metals are the most dangerous components. These comprise combustion particles, abrasion of bearing parts, tire wear, lubricants, residues from weathered paint, and corroded components^57–59^. Other elements may be suspended, transported and deposited by air currents and eventually mix with other solid particles^60,61^. In urban sinkholes in Cancun, the disassembling of electronic wastes is an important potential contamination source; refrigerators, televisions, radios, cellular phones and batteries, which are clearly anthropogenic wastes, have been found in these sites during the cleaning campaigns organized by the local government^62^.

Table 5 presents metal concentrations for some sites in southeastern Mexico with characteristics similar to Cancun’s. Even if values such as those for urban dust or agricultural lands are not quantified, it is worrisome to find contaminated sediments in young urban areas with no industries. These findings add to imminent risks to environmental health (Table 6), and it is important to monitor heavy metal contents in the environment because their presence is related to human health conditions and several diseases.

**Table 5:** Metal concentrations (mg/kg dry weight) in rocks, sediments, soils and urban dust in cities in southeastern Mexico and comparison with international standards.

Sample (Country)	Zn	Fe	Al	Cu	Ni	Pb	Refs.
Calcite/Aragonite fresh aggregate	3–4					3–4	^63^
Carbonate rocks (bulk composition), Yucatan peninsula (Mexico)		249	716	1.71	1.11	1.13	^64^
Wetland surface sediment, Quintana Roo (Mexico)	202 ± 264						^65^
Lake surface sediment, Quintana Roo (Mexico)	0–209	0.5–6%		0–81	0–447	0–41	^66^
Coastal lagoon sediment, Quintana Roo (Mexico)	41–2661	12–1386	69–9425	2.6–50	0.1–5.5	1.1–1.4	^67^
Sediments, Quintana Roo (Mexico)	9.8	731.0		2.0		3.4	^68^
Lake sediments, Quintana Roo (Mexico)	1.36–24.48			1.78–80.39		8.61–45.6	^69^
Core sediment (15–30 cm depth), Yucatán (Mexico)						3–10	^70^
Coastal lagoon sediments, Yucatan (México)	25.3–38.7	1.3–2.0		2.1–16.9	7.6–11.1	0.1–10.6	^71^
Agricultural soil, Quintana Roo (Mexico)		27,663.4		10.5	7.5		^72^
Agricultural soil, Campeche (Mexico)	54.1 ± 12.1	598 ± 223	143 ± 33	24.9 ± 12.3		0.65 ± 0.2	^73^
Soil, Tabasco (Mexico)	6.0	607.6		4.5	7.5	0.6	^74^
Urban dust, Quintana Roo (Mexico)		4240–11,758		21.4–96	21.5–37	23.8–257	^75^
Urban soil, Florida (USA)	32.6–70.9			5.3–19.9	2.2–8.6	18.7–86.3	^76^
Urban soil (southwest China)	373.0			68.6	62.5	68.6	^77^
Sediments (USA)	7–38	0.9–1.8%	26%	-10–25	9.9	4–17	^78^
Sediments (Canada)	123.0			35.7		35.0	^79^
Soil (Finland)	400.0			200.0	150.0	750.0	^80^
Urban sinkholes, Quintana Roo (Mexico)	873.3	1502.6	3184.0	26.9	5.2	9.6	This study

Values represent the average (± standard deviation) or range of reported quantities.

**Table 6:** Health risk and possible sources for human intake of the metals zinc, aluminum, copper, nickel and lead.

	Health risk	Source for human intake	References
Zn	Stomach cramps, nausea, vomiting. Ingesting high levels for several months may cause anemia, damage the pancreas and decrease levels of high density lipoprotein (HDL)	Food and water containing Zn, pharmaceutical and cosmetic products. Inhaled from coal burning, dust or fumes from smelting or welding operations	^50,81^
Al	Lungs and nervous system sensitivity or toxicity following inhalation exposure	Atmospheric aluminum in urban and industrial location, cookware, pharmaceutical products	^81–83^
Cu	Gastrointestinal and hepatic sensitivity or toxicity	Contaminated drinking water, used as antimicrobial agents in drinking water treatments. Household (construction) and electrical material, cookware, fertilizers, wood preservatives, roofing and marine antifouling paints. Cu alloys used in heating, ventilation, and air-conditioning	^81,84^
Ni	Allergic reactions (approximately 10–20% of the population is sensitive to Ni). Chronic bronchitis, reduced lung function, cancer	Inhaled from fumes from trash incinerators. Dermal contact with contaminated soil	^81,84^
Pb	Due to its high molecular weight and chemical characteristics, it competes with and obstructs metabolic processes, and alters the integrity of the cell membranes, the metabolism of vitamin D, DNA transcription, among others. It acts on the brain and the peripheral nervous system. Toxic to kidney and circulatory system, and alters reproductive system and the normal conformation of the fetus	Mainly related to gasoline that contained lead (phased out at the beginning of the 1990s). Glazed ceramic, paints for domestic and outdoor use, mineral mills and metallurgical smelters. Released from recycling lead acid batteries. Maternal–fetal transmission of bone lead from mother to fetus	^85^

## Conclusions

The most abundant metal in the urban sinkholes in Cancun is zinc, both based on concentration and because it was extracted in all the phases of the sequential extraction, which makes it the most available metal to the environment. Pb and Ni were quantified in lower concentrations and are the least available, but they are found in the oldest sites in the city (52 years); thus, their presence could be attributed to the early stages of urbanization. Fe and Al are within the natural ranges found in the soil. Based on the metals found and their concentrations, the main contamination sources seem to be atmospheric deposition and surface runoff, likely from the influence of road traffic and urban dust. In general, the city of Cancun has a low contamination status for heavy metals in the soil after 50 years of existence. Correlations were observed between the degree of urbanization of the city and the degree of pollution, and the trend was greater in the older sections of the city. Given that a high percentage of the metals were extracted in the organic matter phase, land use changes in these sinkholes might result in the release some of these metals into the environment. With these results and the risk assessment presented, we aim to contribute to environmental management in the study area, and the results can be compared with those of other growing tourist cities and other cities with sinkholes around the world.

All data generated or analyzed during this study are included in this published article as Supplementary Tables S1 and S2.

## Supplementary Information

Supplementary Figure S1.

Supplementary Tables.
