# Supplementary figures and images for: Heavy metals in the sediments of urban sinkholes in Cancun, Quintana Roo
Source: Sci Rep. 2023 Apr 29;13:7031. doi: 10.1038/s41598-023-34218-4 (PMC10148832; doi:10.1038/s41598-023-34218-4)

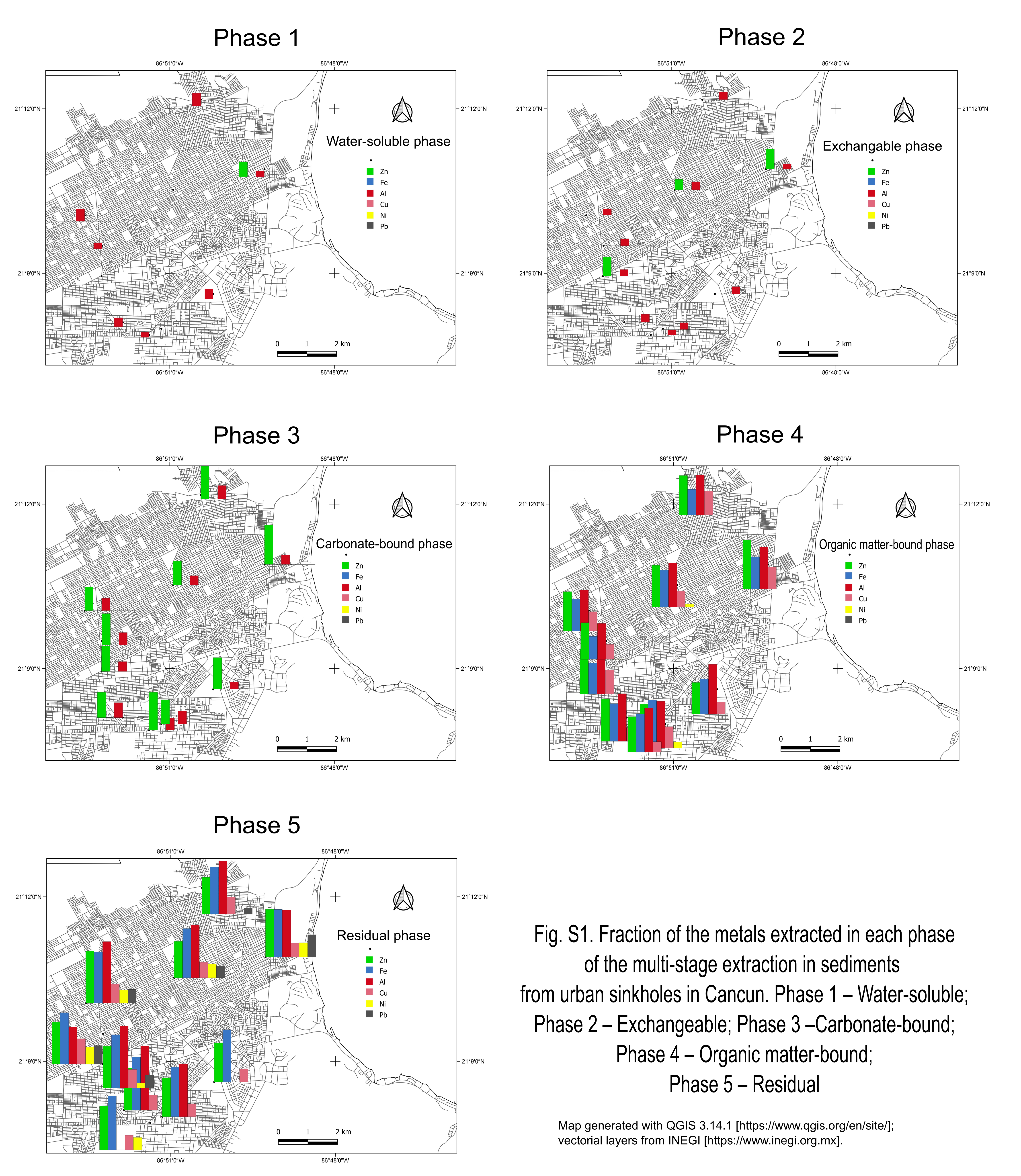

Supplement: Supplementary file 1 — Supplementary Figure S1. [file 41598_2023_34218_MOESM1_ESM.png]
